# Assisted Reproductive Technology and Newborn Size in Singletons Resulting from Fresh and Cryopreserved Embryos Transfer

**DOI:** 10.1371/journal.pone.0169869

**Published:** 2017-01-23

**Authors:** Galit Levi Dunietz, Claudia Holzman, Yujia Zhang, Nicole M. Talge, Chenxi Li, David Todem, Sheree L. Boulet, Patricia McKane, Dmitry M. Kissin, Glenn Copeland, Dana Bernson, Michael P. Diamond

**Affiliations:** 1 Department of Neurology, University of Michigan, Ann Arbor, MI, United States of America; 2 Department of Epidemiology and Biostatistics, Michigan State University, East Lansing, MI, United States of America; 3 Division of Reproductive Health at the Centers for Disease Control and Prevention, Atlanta, GA, United States of America; 4 Michigan Department of Health and Human Services, Maternal and Child Health Epidemiology Section, Lansing, MI, United States of America; 5 Michigan Department of Health and Human Services, Division for Vital Records and Health Statistics, Lansing, MI, United States of America; 6 Massachusetts Department of Public Health, Boston, MA, United States of America; 7 Department of Obstetrics and Gynecology, Augusta University, Augusta, GA, United States of America; Universite Blaise Pascal, FRANCE

## Abstract

**Objectives and Study Design:**

The aim of this study was two-fold: to investigate the association of Assisted Reproductive Technology (ART) and small newborn size, using standardized measures; and to examine within strata of fresh and cryopreserved embryos transfer, whether this association is influenced by parental infertility diagnoses. We used a population-based retrospective cohort from Michigan (2000–2009), Florida and Massachusetts (2000–2010). Our sample included 28,946 ART singletons conceived with non-donor oocytes and 4,263,846 non-ART singletons.

**Methods:**

Regression models were used to examine the association of ART and newborn size, measured as small for gestational age (SGA) and birth-weight-z-score, among four mutually exclusive infertility groups: female infertility only, male infertility only, combined female and male infertility, and unexplained infertility, stratified by fresh and cryopreserved embryos transfer.

**Results:**

We found increased SGA odds among ART singletons from fresh embryos transfer compared with non-ART singletons, with little difference by infertility source [adjusted odds-ratio for SGA among female infertility only: 1.18 (95% CI 1.10, 1.26), male infertility only: 1.20 (95% CI 1.10, 1.32), male and female infertility: 1.18 (95% CI 1.06, 1.31) and unexplained infertility: 1.24 (95% CI 1.10, 1.38)]. Conversely, ART singletons, born following cryopreserved embryos transfer, had lower SGA odds compared with non-ART singletons, with mild variation by infertility source [adjusted odds-ratio for SGA among female infertility only: 0.56 (95% CI 0.45, 0.71), male infertility only: 0.64 (95% CI 0.47, 0.86), male and female infertility: 0.52 (95% CI 0.36, 0.77) and unexplained infertility: 0.71 (95% CI 0.47, 1.06)]. Birth-weight-z-score was significantly lower for ART singletons born following fresh embryos transfer than non-ART singletons, regardless of infertility diagnoses.

## Introduction

Assisted Reproductive Technology (ART) is an infertility therapy that involves the handling of both gametes in the laboratory to achieve pregnancy. ART-conceived singletons have an increased risk for low birth weight (LBW) compared with singletons in the general population.[1–9] LBW has long been used as an indicator for child health, however, its interpretation is unclear because LBW may be related to short gestation, small newborn size or their combination.[10–12] Therefore, indicators that distinguish LBW infants resulting from short gestation or small newborn size provide a more informative measure of risk by reducing this confounding. Two such indicators are small for gestational age (SGA) and birth-weight-z-score.

The definition of SGA varies across studies and may include infants whose birth weight is below the 10^th^ or 5^th^ percentile (SGA/10^th^ or SGA/5^th^) for gestational age or whose birth weight is ≥2 standard deviations below the mean birth weight for gestational age. Two recent studies did not detect an increased risk of SGA/10^th^ for ART compared with non-ART singletons[13, 14], whereas, other studies reported a significantly increased risk of SGA/10^th^ among ART singletons, with odds ratios ranging from 1.22–2.29.[8, 15–17] Using the 5^th^ percentile, ART singletons were found to have a 40% higher odds for SGA birth compared with their non-ART counterparts.[18] These conflicting results may be attributed to the variety of SGA definitions, or to other sources of heterogeneity such as sample size and/or the approach to potential confounders e.g. plurality, social factors, ART and infertility characteristics.

Birth-weight-z-score, constructed as a continuous measure, allows the comparison of newborn size across gestational ages, sexes and birth weights, representing the same *relative* birth weight for infants, rather than their *absolute* weight. Compared with LBW, newborn size measured as birth-weight-z-score has rarely been used to investigate birth outcomes in ART populations.[19]

Previous reports suggested differential birth weight for ART singletons conceived with fresh versus cryopreserved embryos, with smaller newborn size for the former. [20–22] In contrast, one cohort study associated lower birth weight with ART singletons from cryopreserved embryos transfer. [23] The inconsistent findings across studies of ART and newborn size warrant additional investigation. The purpose of this study was to use data from the States Monitoring Assisted Reproductive Technology (SMART) Collaborative to examine: 1) whether ART singletons, born following a fresh or cryopreserved embryos transfer, are at higher risk of small newborn size, measured by both SGA/10^th^, SGA/5th and birth-weight-z-score, compared with singletons in the general population; and 2) whether an association between ART and newborn size is driven by infertility source (female, male, combined, or unexplained infertility).

## Materials and Methods

### Study Population

We used a population-based dataset of birth certificates from three states linked to the National ART Surveillance System (NASS) by the SMART Collaborative project. The SMART Collaborative was established by the Centers for Disease Control and Prevention (CDC) and public health agencies of Florida, Massachusetts and Michigan to evaluate maternal and perinatal outcomes of ART and to improve state-based ART surveillance.[24] The sample included all live births in Michigan from 2000–2009 and in Florida and Massachusetts from 2000–2010, linked to ART cycles in the respective states using a probabilistic linkage method with a high linkage rate (87.8%) and good validity.[25]

**Fig 1:**
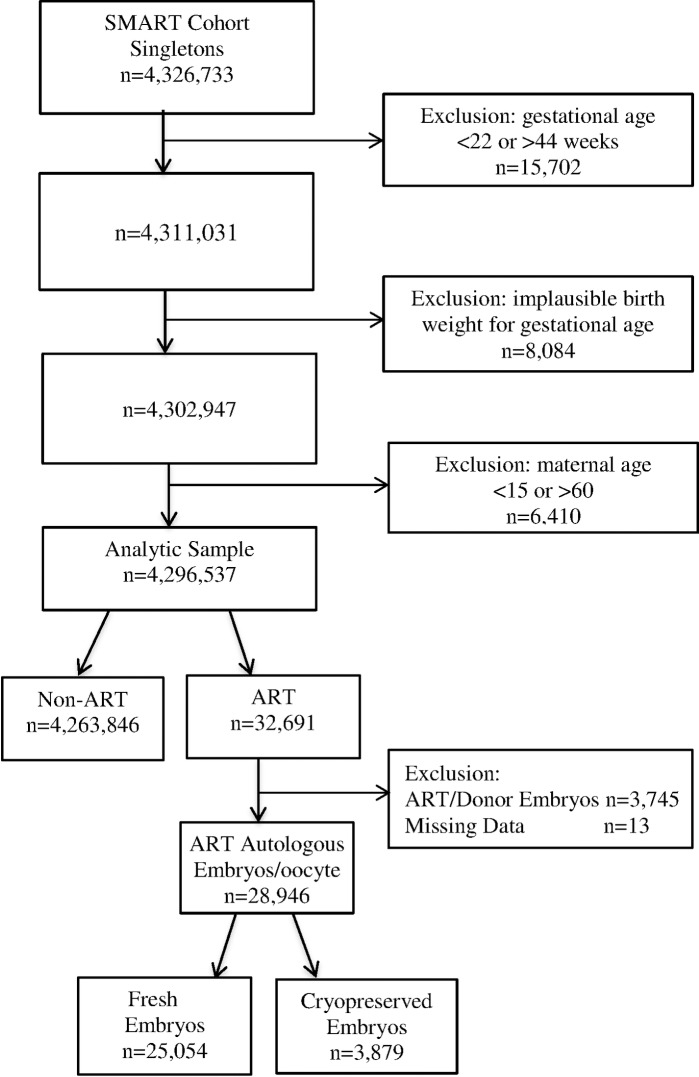
Flowchart of Participants in the States Monitoring Assisted Reproductive Technology (SMART) dataset: Florida and Massachusetts 2000–2010 and Michigan 2000–2009.

We restricted the sample to singletons born to mothers aged 15–60 between 22 and 44 weeks’ gestation. We then excluded records with implausible combinations of birth weight and gestational age using the approach described in Table 1 of Alexander et al [26] along with recently published criteria. [27, 28] Finally, we excluded singletons, conceived with donor oocytes from the ART group and created two strata of ART singletons, born following a fresh or cryopreserved embryos transfer. After all exclusions, the final dataset included a total of 4,292,792 singleton live births, of which 4,263,846 (99%) were non-ART and 28,946 (1%) were ART related births. ART singletons included 25,054 infants from fresh embryos transfer and 3,879 from cryopreserved embryos transfer (Fig 1).

The study received approval from the Institutional Review Boards of Florida, Massachusetts, Michigan and the CDC.

### Infertility Groups

Infertility diagnoses for ART users were obtained from the NASS data. We further divided the ART singletons, conceived with non-donor oocytes, into four mutually exclusive subgroups based on their parental infertility diagnosis: female infertility only, male infertility only, combined male and female infertility, and unexplained infertility. Finally, each singleton within the infertility subgroup was assigned to one of two strata, based on whether their birth resulted from fresh or cryopreserved embryos transfer. Women were classified in the non-ART group if their birth record was not matched to the NASS data. NASS entries that were not linked to birth certificate were excluded.

### Newborn Size

Clinically estimated gestational age, birth weight, and sex, were abstracted from birth certificates. We then used this information in conjunction with a sex- and gestational age- specific population-based reference to identify SGA births and generate birth weight z-scores. [28] This birth weight reference is based upon singleton live births between 22–44 weeks to United States resident women in 2009–2010. Unlike previous United States population-based references, this reference provides the information needed to generate both categorical (percentile thresholds) and continuous measures of birth size (means, standard deviations for z-score calculations). We defined SGA according to two different thresholds (SGA/10^th^ and SGA/5^th^, respectively), with non-SGA as the referent.

### Covariates

Maternal education, race, age at the time of delivery, parity, state of residence and delivery year were included in the adjusted models, based on prior knowledge from the scientific literature and their statistical significance in univariate models. To meet requirements of small cell sizes in contingency tables, we collapsed categories of race/ethnicity and education in the adjusted analyses. Specifically, we collapsed race/ethnicity categories of ‘Asian/Pacific Islander or other’ into one group, and education categories of ‘high school/GED diploma’ and ‘less than high school’ into one group.

### Statistical Analysis

We used basic descriptive statistics, chi-square tests and linear regression to compare the distributions of maternal and infant characteristics among ART and non-ART groups as well as before and after excluding those with implausible birth weight and gestational age combinations (see *Study Population*). To evaluate the associations with newborn size, SGA and birth–weight-z-score, among ART singletons from fresh or cryopreserved embryos transfer, we constructed logistic and linear regression models, respectively, using non-ART infants as the referent group. We applied robust variance estimators to address the correlation between infants delivered by the same mother during the study period, given that the unit of analysis was a live birth. In the adjusted models we included parity, maternal age, race/ethnicity, education, state of residence and delivery year as the covariates. Body mass index (BMI) information was not available for all states across the study period, thus was not included in the adjusted model.

ART singletons may have originated from multifetal pregnancies and a subsequent loss of a co-twin.[29, 30] To investigate the influence of early fetal loss on newborn size of the surviving singleton, we excluded all singleton births with more than one embryo observed by an ultrasound at six weeks’ gestation.

Finally, we repeated the analyses of SGA/10^th^ and ART after removing preterm births. The construct of SGA/10^th^ for preterm infants has some inherent bias because infants born preterm tend to be smaller than their counterparts who remain in-utero. By comparing only full-term ART and non-ART, we examined the robustness of the results absent this potential preterm bias.

SAS 9.3 (Cary, NC) was used for logistic models analysis. Linear regression models that compared birth-weight-z-scores across study groups were generated with PROC REGRESS in SUDAAN 11 (Research Triangle Park, NC) statistical software.

## Results

After excluding infants with implausible combinations of birth weight and gestational age, the final sample included 4,292,792 infants. Frequencies of maternal and infant characteristics were similar in samples with and without the excluded births. In descriptive analyses (Table 1), ART mothers were significantly older, more educated and more likely to be Non-Hispanic white and primiparous compared with non-ART mothers. Infants born to non-ART and ART mothers had a similar sex distribution, but ART infants’ mean birth weight was 24 grams lower. Compared with non-ART singletons, all three measures of newborn size (SGA/10^th^, SGA/5^th^, and birth-weight-z-score), were similar among ART singletons born following a fresh embryos transfer (p>0.05), but different for ART births from cryopreserved embryos transfer.

**Table 1:** Maternal and infant characteristics for fresh and frozen autologous ART cycles and non-ART singleton live births in Massachusetts and Florida 2000–2010 and in Michigan 2000–2009: After exclusion of implausible birth weight for gestational age.

Maternal, infant Characteristics	Non-ART	ART	P value †
Sample Size N (%) ‡	4,263,846 (99)	32,691 (<1)	<0.01
Maternal Age (mean)	27.7	35.5	<0.01
Maternal Race/Ethnicity			
Non-Hispanic White	2,447,489 (58)	26,136 (81)	
Non-Hispanic Black	759,051 (18)	1,263 (4)	<0.01
Hispanic	803,781 (19)	2,798 (9)	
Asian/Other	222,012 (5)	2,012 (6)	
Maternal Education			
High school or lower	2,042,263 (48)	4,230 (13)	
Some college	1,044,839 (25)	6,747 (21)	<0.01
Bachelor’s or higher	1,138,459 (27)	21,538 (66)	
Parity			
0	1,794,285 (42)	21,284 (65)	
1	1,392,288 (33)	8,446 (26)	<0.01
2	669,158 (16)	2,046 (6)	
≥3	408,115 (10)	915 (3)	
Newborn Sex			
Male	2,184,140 (51)	16,770 (51)	0.79
Female	2,079,706 (49)	15,921 (49)	
Mean Birth weight (g) (se)	3,320 (0.3)	3,296 (3.4)	<0.01
SGA <10^th^ percentile	400,220 (9.4)	Fresh 2,324 (9.3)	0.55
		Frozen 240 (8.3)	0.03
SGA <5^th^ percentile	193,192 (4.5)	Fresh 1,116 (4.5)	0.56
		Frozen 114 (3.9)	0.09
Birth weight z-Score (se)	0.03 (0.0005)	Fresh 0.031 (0.0065)	0.78
		Frozen 0.103 (0.0187)	<0.01

ART = Assisted reproductive technology.

†P values computed for correlated data.

‡ some columns may not add to the total sample size, due to missing values in some categories.

The associations between ART treatment and newborn size (SGA/10^th^ and SGA/5^th^) for the pooled ART group and for each infertility subgroup, stratified by fresh and cryopreserved embryos transfer, are presented in Table 2. In adjusted analyses, the odds of an SGA/10^th^ or SGA/5^th^ infant from fresh embryos transfer were significantly greater in the ART combined group than in the non-ART group [adjusted odds ratio (aOR) 1.19 (95% CI 1.14, 1.25) and aOR 1.17 (95% CI 1.10, 1.25), respectively]. Each ART subgroup had increased odds of delivering a SGA/10^th^ infant relative to the non-ART group. Similar results were observed in adjusted SGA/5^th^ models with the exception of male infertility diagnosis. Conversely, ART infants born following a cryopreserved embryos transfer were less likely to be SGA/10^th^ or SGA/5^th^ compared with non-ART singletons.

**Table 2:** Associations between newborn size and ART singletons born following fresh or cryopreserved embryos transfer, using non-ART as the referent group. Population-based data of all singleton live births in Florida and Massachusetts 2000–2010 and in Michigan 2000–2009.

Fresh Embryos Transfer							
ART/Infertility Type	N			Small for Gestational Age †			
				10^th^ percentile		5^th^ percentile	
	Total (%)	SGA <10^th^	SGA <5^th^	cOR (95% CI)	‡ aOR (95% CI)	cOR (95% CI)	‡ aOR (95% CI)
Non-ART	4,263,846 (>99)	400,220	193,192	Reference	Reference	Reference	Reference
ART (all users)	25,054	2,324	1,116	0.99 (0.95, 1.03)	1.19 (1.14, 1.25)	0.98 (0.92, 1.04)	1.17 (1.10, 1.25)
ART/Female	11,086	1,029	489	0.99 (0.93, 1.05)	1.18 (1.10, 1.26)	0.97 (0.89, 1.07)	1.14 (1.04, 1.25)
ART/Male	5,952	548	239	0.98 (0.90, 1.07)	1.20 (1.10, 1.32)	0.88 (0.77, 1.00)	1.09 (0.95, 1.24)
ART/Combined	4,176	389	211	0.99 (0.89, 1.10)	1.18 (1.06, 1.31)	1.12 (0.98, 1.29)	1.32 (1.14, 1.52)
ART/Unexplained	3,840	358	177	0.99 (0.89, 1.11)	1.24 (1.10, 1.38)	1.02 (0.87, 1.19)	1.24 (1.06, 1.45)
Cryopreserved Embryos Transfer							
ART/Infertility Type	N			Small for Gestational Age †			
				10^th^ percentile		5^th^ percentile	
	Total (%)	SGA <10^th^	SGA <5^th^	cOR (95% CI)	‡ aOR (95% CI)	cOR (95% CI)	‡ aOR (95% CI)
Non-ART	4,263,846 (>99)	400,220	193,192	Reference	Reference	Reference	Reference
ART (all users)	3,879	182	83	0.48 (0.41, 0.55)	0.59 (0.51, 0.69)	0.46 (0.37, 0.57)	0.56 (0.44, 0.70)
ART/Female	1,771	82	39	0.47 (0.38, 0.59)	0.56 (0.45, 0.71)	0.47 (0.35, 0.65)	0.53 (0.38, 0.75)
ART/Male	943	47	19	0.51 (0.38, 0.68)	0.64 (0.47, 0.86)	0.43 (0.28, 0.68)	0.54 (0.34, 0.87)
ART/Combined	681	28	12	0.41 (0.28, 0.60)	0.52 (0.36, 0.77)	0.38 (0.21, 0.67)	0.48 (0.27, 0.85)
ART/Unexplained	484	25	13	0.53 (0.35, 0.79)	0.71 (0.47, 1.06)	0.58 (0.34, 1.01)	0.78 (0.45, 1.36)

ART = assisted reproductive technology; SGA = small for gestational age; cOR = crude odds ratio; aOR = Adjusted odds ratio; CI = Confidence interval.

†Sex specific.

‡ Adjusted for parity, age, race and education level, state of residence and delivery year.

After excluding all preterm births from the cohort, the statistically significant association between ART and small newborn size remained. Full term ART singletons from fresh embryos transfer were more likely to be SGA/10^th^, while full term singletons from cryopreserved embryos transfer were less likely to be born SGA/10^th^, relative to full term non-ART singletons [aOR 1.22 (95% CI 1.17, 1.28)] and [aOR 0.60 (95% CI 0.51, 0.71)] respectively. Similar results were observed when each ART subgroup was compared with the non-ART group (Table 3).

**Table 3:** Associations between full-term newborn size and ART singletons born following fresh or cryopreserved embryos transfer, using non-ART as the referent group. Population-based data of all singleton live births in Florida and Massachusetts 2000–2010 and in Michigan 2000–2009.

Fresh Embryos Transfer / Full Term Singletons				
ART/Infertility Type	N		Small for Gestational Age– 10^th^ percentile †	
	Total (%)	SGA <10^th^	cOR (95% CI)	‡ aOR (95% CI)
Non-ART	3,921,491 (>99)	364,199	Reference	Reference
ART (all users)	22,306	2,044	0.99 (0.94, 1.03)	1.22 (1.17, 1.28)
ART/Female	9,728	885	0.98 (0.91, 1.05)	1.20 (1.11, 1.29)
ART/Male	5,403	488	0.97 (0.88, 1.07)	1.22 (1.11, 1.34)
ART/Combined	3,703	352	1.03 (0.92, 1.15)	1.25 (1.11, 1.41)
ART/Unexplained	3,472	319	0.99 (0.88, 1.11)	1.27 (1.13, 1.43)
Cryopreserved Embryos Transfer / Full Term Singletons				
ART/Infertility Type	N		Small for Gestational Age– 10^th^ percentile †	
	Total (%)	SGA <10^th^	cOR (95% CI)	‡ aOR (95% CI)
Non-ART	3,921,491 (>99)	364,199	Reference	Reference
ART (all users)	3,456 (<1)	160	0.47 (0.40, 0.56)	0.60 (0.51, 0.71)
ART/Female	1,580	69	0.45 (0.35, 0.57)	0.55 (0.43, 0.70)
ART/Male	862	43	0.51 (0.38, 0.70)	0.65 (0.48, 0.90)
ART/Combined	586	25	0.44 (0.29, 0.45)	0.56 (0.37, 0.84)
ART/Unexplained	428	23	0.55 (0.36, 0.85)	0.76 (0.50, 1.16)

ART = assisted reproductive technology; SGA = small for gestational age; cOR = crude odds ratio

aOR = Adjusted odds ratio; CI = Confidence interval.

† Sex specific.

‡ Adjusted for parity, age, race and education level, state of residence and delivery year.

Next, newborn size was modeled as a continuous variable (birth-weight-z-score) with non-ART singletons as the referent group (Table 4). We observed that all ART singleton births from fresh embryos had a negative mean birth-weight-z-score, i.e., their mean birth weight was below the mean of the reference population. In contrast, non-ART singleton and ART singletons from cryopreserved embryos transfer, had mean birth-weight-z-scores above that of the reference population.

**Table 4:** Associations between birth weight z-score and ART singletons born following fresh or cryopreserved embryos transfer, using non-ART as the referent group. Population-based data of all singleton live births in Florida and Massachusetts 2000–2010 and Michigan 2000–2009.

Fresh Embryos					
ART/Infertility Type	N (%)	Birth Weight z-score † Crude (CI)	Birth Weight z-score † Adjusted ‡ (CI)	Regression coefficient Adjusted ‡ (CI)	P Value ‡
Non-ART	4,263,846 (>99)	0.03 (0.03, 0.03)	0.03 (0.03, 0.04)	Reference	Referent
ART/Female	11,086	0.03 (0.01, 0.05)	-0.08 (-0.10, -0.06)	-0.11 (-0.13, -0.09)	<0.01
ART/Male	5,952	0.04 (0.02, 0.07)	-0.06 (-0.09, -0.04)	-0.10 (-0.12, -0.07)	<0.01
ART/Combined	4,176	0.03 (-0.01, 0.06)	-0.07 (-0.10, -0.04)	-0.11 (-0.14, -0.08)	<0.01
ART/Unexplained	3,840	0.03 (-0.01, 0.06)	-0.09 (-0.12, -0.06)	-0.13 (-0.16, -0.09)	<0.01
Cryopreserved Embryos					
ART/Infertility Type	N (%)	Birth Weight z-score † Crude (CI)	Birth Weight z-score † Adjusted ‡ (CI)	Regression coefficient Adjusted ‡ (CI)	P Value ‡
Non-ART	4,263,846 (>99)	0.03 (0.03, 0.03)	0.03 (0.03, 0.03)	Reference	Referent
ART/Female	1,771	0.38 (0.33, 0.43)	0.26 (0.21, 0.31)	0.22 (0.17, 0.27)	<0.01
ART/Male	943	0.37 (0.31, 0.44)	0.26 (0.19, 0.32)	0.22 (0.10, 0.29)	<0.01
ART/Combined	681	0.33 (0.25, 0.40)	0.21 (0.14, 0.29)	0.18 (0.10, 0.26)	<0.01
ART/Unexplained	484	0.35 (0.26, 0.44)	0.19 (0.10, 0.29)	0.16 (0.07, 0.25)	<0.01

ART = assisted reproductive technology; CI = confidence interval.

† Gestational age and sex specific.

‡ Adjusted for parity, age, race and education level, state of residence and delivery year.

## Discussion

We investigated the association between ART and newborn size using one of the largest datasets of ART data linked to birth records, involving over four million newborns. SGA and birth-weight-z-scores are informative outcomes and more specific to newborn size compared with other frequently used measures, e.g. LBW and mean birth weight. Our findings suggest that ART singletons from fresh embryos transfer had increased odds of being SGA/10^th^ regardless of whether infertility was diagnosed in the female patient, male partner, both, or was unexplained. Conversely, ART singletons, resulted from cryopreserved embryos transfer, had lower odds of being SGA/10^th^ compared with singletons in the general population.

Our observed SGA/10^th^ odds for ART singletons born after fresh embryos transfer relative to non-ART singletons were more modest in comparison to a recent Australian study that reported a 1.5-fold higher SGA/10^th^ risk among ART singletons from fresh embryos transfer compared with non-ART singleton infants. Our results for SGA/10^th^ among ART singletons from cryopreserved embryos transfer compared with non-ART singletons were aligned with their findings. [16] In other studies, when ART singletons from fresh embryos transfer were selected as the reference group, ART singletons from cryopreserved embryos transfer had lower [20, 23] or the same odds [31] for SGA/10^th^. This is consistent with reports that large for gestational age and macrosomic singletons have been associated with transfer of cryopreserved embryos compared with singletons from fresh embryos transfer or non-ART singletons. [31, 32]

Inconsistent results across studies of ART and newborn size of singletons from fresh or cryopreserved embryos transfer might be explained by multiple heterogeneous elements, such as SGA definitions, SGA/10^th^, or SGA< 2 standard deviations below a growth standard of a reference population, [16, 31] selection of different control groups, [16, 23] or lack of adjustment for important confounders, such as parity or maternal age. [20] In this analyses, we included parity as a confounder to allow inclusion of females with primary infertility as well as women with potentially secondary infertility, who otherwise would have been excluded from this study. Thus, the current effects reflect a comprehensive and diverse population of subfertile ART users.

Post hoc univariate analyses showed that although all covariates were statistically significant, only ‘maternal education’ and ‘maternal age’ contributed largely to the risk of small newborn size among ART singletons. These factors were previously reported as risk factors for small size at birth.[33, 34]

Although birth-weight-z-scores are recommended for reporting perinatal outcomes among ART populations [19, 35] such studies are rare. In 2008, Shih et al, used birth-weight-z-scores based on British growth reference data to examine whether newborn size was associated with different types of ART treatment.[21] While their findings suggested lower mean birth-weight-z-scores among infants born to couples who used ART with fresh embryos, -0.163 (SD = 1.004) compared with infants of non-ART couples -0.061 (SD = 1.099), both groups had mean birth-weight-z-scores below the expected mean. In this analysis we used two indicators, SGA and birth-weight-z-score, to measure sex-specific newborn size both categorically and continuously. To minimize measurement errors, both indicators were carefully constructed after exclusion of birth records with implausible birth weight and gestational age combinations using established and recently published criteria and algorithms.[26–28] In contrast to Shih, [21] our results showed that the non-ART group had mean birth-weight-z-scores slightly above that of the reference population, whereas the ART group’s means of singletons from fresh embryos transfer were below the referent.

The differential newborn size of ART singletons from fresh or cryopreserved embryos transfer is poorly understood, and may be partially explained by aspects related to ART procedure and patient profiles. ART cycles involving cryopreserved embryos transfer are associated with reduced ovarian stimulation, thus, improved endometrial receptivity, no oocyte retrieval and transfer of higher quality embryos that survived the freezing-thawing process. [23, 36] However, recent data suggest that the freezing-thawing process complicates ART pregnancies with preeclampsia. [37, 38]

In this study design and analyses we tried to address limitations of previous studies, however, some limitations remain. Although SGA is a better indicator of newborn size than LBW, it still represents a heterogeneous group, i.e. those who are constitutionally small and those with pathologically small newborn size. In addition, fetuses who begin as appropriate size and then experience poor growth may not meet the 10^th^ percentile cutoff at birth, thus using SGA as an outcome could result in some misclassification of small newborn size for infants above and below the cut point. [39] SGA is constructed using population-based references of birth weight while excluding fetal size of unborn fetuses. As a result, SGA formulation for preterm infants may be prone to bias resulting from birth weight differences between preterm infants (often smaller) and those who remain in-utero. In an attempt to minimize this observational bias, we repeated the analysis using only full term infants. The results indicated a similar risk of smaller newborn size for ART singletons, from fresh or cryopreserved embryos transfer, versus non-ART infants born at term. Potential confounders such as BMI, smoking and an early loss of a co-twin were not included in the primary analyses. BMI information was not available for all states and years. Although smoking is associated with small newborn size [40], it was excluded from the primary analyses due to its low prevalence (1%) among ART mothers and its low reliability in birth records. [41, 42] A secondary analysis with smoking included in the adjusted regression models produced similar effect estimates among all ART subgroups. Our results were also unaffected when analyses were restricted to singleton births originating from single fetal pregnancies. Treatment information on culture media, freezing techniques, day of embryo transfer and pre-implantation genetic diagnosis (PGD) were not available for the years included in this study. In 2011–2012, PGD was reported in 4.5% of fresh embryos transfer [43] and is therefore unlikely to have a significant impact on our findings.

The SMART dataset includes two distinct populations, ART and non-ART couples. We recognize that there are unidentified couples with infertility among non-ART users. These subfertile couples may have conceived using non-ART therapy to treat subfertility or achieved a spontaneous pregnancy. The resulting misclassification could have attenuated the association between ART births and small newborn size. We also acknowledge that data quality with respect to timing of conception may vary by the mode of conception, and is more accurate for the ART population. To create the SMART dataset, a probabilistic method was used to link birth certificates and ART surveillance data. While highly successful, with reported linkage rate of 87.8% and a good validity, this method is not free of matching errors.[25] In 2010, it was estimated that ART surveillance data represents more than 97% of ART cycles performed in the US, as only few small clinics did not provide their data to NASS.[44]

## Conclusions

We found that the risk of smaller newborn size was higher in ART singletons from fresh embryos transfer and lower in ART singletons from cryopreserved embryos transfer compared with non-ART singleton infants. Although statistically significant, the magnitude of excess risk among ART singletons from fresh embryos transfer was small, which is reassuring. Similarly, the excess risk of smaller newborn size within ART subgroups defined by infertility source (male, female), was not large. Greater subgroup heterogeneity in newborn size may be detected if assessed by underlying infertility causes and ART therapy subtypes; our future work will investigate this potential heterogeneity.
