# Correction: Assisted Reproductive Technology and Newborn Size in Singletons Resulting from Fresh and Cryopreserved Embryos Transfer

**DOI:** 10.1371/journal.pone.0196767

**Published:** 2018-05-10

**Authors:** Galit Levi Dunietz, Claudia Holzman, Yujia Zhang, Nicole M. Talge, Chenxi Li, David Todem, Sheree L. Boulet, Patricia McKane, Dmitry M. Kissin, Glenn Copeland, Dana Bernson, Michael P. Diamond

The first author’s name appears incorrectly in the citation. The first author’s name in the citation should be Dunietz GL. The correct citation is: Dunietz GL, Holzman C, Zhang Y, Talge NM, Li C, Todem D, et al. (2017) Assisted Reproductive Technology and Newborn Size in Singletons Resulting from Fresh and Cryopreserved Embryos Transfer. PLoS ONE 12(1): e0169869. doi:10.1371/journal.pone.0169869
